# Cannabinoid-hypocretin cross-talk in the central nervous system: what we know so far

**DOI:** 10.3389/fnins.2013.00256

**Published:** 2013-12-20

**Authors:** África Flores, Rafael Maldonado, Fernando Berrendero

**Affiliations:** Laboratory of Neuropharmacology, Department of Experimental and Health Sciences, Universitat Pompeu FabraBarcelona, Spain

**Keywords:** hypocretinergic system, endocannabinoid system, heteromerization, reward, energy balance, antinociception, sleep/wake cycle

## Abstract

Emerging findings suggest the existence of a cross-talk between hypocretinergic and endocannabinoid systems. Although few studies have examined this relationship, the apparent overlap observed in the neuroanatomical distribution of both systems as well as their putative functions strongly point to the existence of such cross-modulation. In agreement, biochemical and functional studies have revealed the existence of heterodimers between CB1 cannabinoid receptor and hypocretin receptor-1, which modulates the cellular localization and downstream signaling of both receptors. Moreover, the activation of hypocretin receptor-1 stimulates the synthesis of 2-arachidonoyl glycerol culminating in the retrograde inhibition of neighboring cells and suggesting that endocannabinoids could contribute to some hypocretin effects. Pharmacological data indicate that endocannabinoids and hypocretins might have common physiological functions in the regulation of appetite, reward and analgesia. In contrast, these neuromodulatory systems seem to play antagonistic roles in the regulation of sleep/wake cycle and anxiety-like responses. The present review attempts to piece together what is known about this interesting interaction and describes its potential therapeutic implications.

## Endocannabinoids and hypocretins: two essential neuromodulators

The extracts of the plant *Cannabis sativa* contain about 60 active compounds of which Δ^9^-tetrahydrocannabinol (THC) is the main psychoactive component (Hall and Degenhardt, 2009). Although it was initially believed that THC exerted its effects by interacting with the plasma membrane due to its high lipophilic nature (Martin, 1986), the site of action of this substance is an endogenous neuromodulatory system termed endogenous cannabinoid system. The endocannabinoid system is constituted by membrane receptors, their fatty-acid derived endogenous ligands and the enzymatic machinery that synthesizes and degrades these lipidic neurotransmitters.

At least two different cannabinoid receptors have been cloned, termed CB1 and CB2 receptors, which share only 44% amino acid (AA) sequence homology (Matsuda et al., 1990; Munro et al., 1993). The distribution of CB1 and CB2 is markedly different: CB1 is the most abundant G-protein coupled receptor (GPCR) in the central nervous system (CNS) (Herkenham et al., 1990), while CB2 is mainly found in immune cells and peripheral tissues (Munro et al., 1993), although some CB2 expression is detected in the brainstem, cortex and cerebellar neurons and microglia (Núñez et al., 2004; Van Sickle et al., 2005). In addition, cannabinoid compounds are able to activate other “non-CB” receptors, such as GPR55, peroxisome proliferator-activated receptors (PPARs), and vanilloid type TRP channels (Pertwee et al., 2010; Kukkonen, 2011).

Endocannabinoids are arachidonic acid-containing messengers generated by phospholipase action, produced on demand at the site of need and are not usually stored in vesicles like classical neurotransmitters (Di Marzo, 2009). The most important endocannabinoids identified are N-arachidonylethanolamine (anandamide) and 2-arachidonylglycerol (2-AG) (Devane et al., 1992; Sugiura et al., 1995), although other ligands such as noladin ether and virodhamide have also been detected (Hanus et al., 2001; Porter et al., 2002). Endocannabinoids are known to act as retrograde regulators of synaptic transmission. Thus, after being synthesized in postsynaptic neurons in response to a depolarization-induced increase in intracellular Ca^2+^, and released to act on CB1 expressed in presynaptic and/or nearby neurons, endocannabinoids attenuate presynaptic depolarization and subsequent neurotransmitter release (Kano et al., 2009). After synthesis and release, endocannabinoid signaling is terminated by reuptake into both neurons and glia followed by intracellular hydrolysis of anandamide and 2-AG, carried out by fatty acid amide hydrolase (FAAH) and monoacylglycerol lipase (MAGL), respectively (Muccioli, 2010).

It has been extensively reported that the endocannabinoid system reciprocally modulates other neurotransmitter systems. The interaction with the endogenous opioid system is the most explored of these cross-talks (Robledo et al., 2008; Parolaro et al., 2010), but pharmacological and biochemical data also reveal an interplay at both molecular and functional levels with other neurotransmitters, such as the dopaminergic and adenosinergic systems (Carriba et al., 2007; Ferré et al., 2009; Fernández-Ruiz et al., 2010). Interestingly, emerging evidence points to a cross-modulation between endocannabinoid and hypocretinergic systems, providing a new range of potential therapeutic applications to currently existing drugs targeting these systems.

Hypocretin-1/orexin-A and hypocretin-2/orexin-B are two neuropeptides proteolytically cleaved from the same precursor, prepro-hypocretin/prepro-orexin (de Lecea et al., 1998; Sakurai et al., 1998). Hypocretin-1 is constituted by 33 AA and post-translationally stabilized by two intrachain disulphide bonds, whereas hypocretin-2 consists of 28 AA and remains as a linear peptide (Sakurai et al., 1998). As other neuromodulatory peptides, hypocretins are stored at the axon terminals within secretory vesicles, which release their content in a Ca^2+^-dependent manner (de Lecea et al., 1998). In contrast to endocannabinoids, hypocretins act mainly as neuroexcitatory modulators. Hypocretins induce neuroexcitation at both pre- and postsynaptic levels: activation of presynaptic Ca^2+^ channels facilitates neurotransmitter release (van den Pol et al., 1998; Li et al., 2002), and regulation of diverse postsynaptic ion channels leads to postsynaptic depolarization (Kukkonen et al., 2002). However, few studies also report hypocretin-induced synaptic inhibition, although the mechanisms underlying this effect remain unclear (Davis et al., 2003; Ma et al., 2007).

So far, two hypocretin receptors with 64% AA sequence homology have been identified: hypocretin or orexin receptor-1 (HcrtR1/OxR1) and hypocretin or orexin receptor-2 (HcrtR2/OxR2) (Sakurai et al., 1998). HcrtR1 displays a 10-fold higher affinity for hypocretin-1 than hypocretin-2, whereas HcrtR2 has equal affinity for both peptides (Smart et al., 2001; Ammoun et al., 2003). Hypocretin-expressing neurons are located exclusively in the hypothalamus, especially in the lateral (LH), perifornical (PFA), and dorsomedial areas (DMH) (de Lecea et al., 1998; Peyron et al., 1998; Sakurai et al., 1998). Despite representing a relatively small population of cells, hypocretinergic neurons send projections widely through the entire neuroaxis of the CNS (Peyron et al., 1998), suggesting that hypocretins modulate the activity of multiple neurotransmitter systems and therefore regulate diverse physiological functions.

## Common characteristics between endocannabinoid and hypocretinergic systems

**Figure 1:**
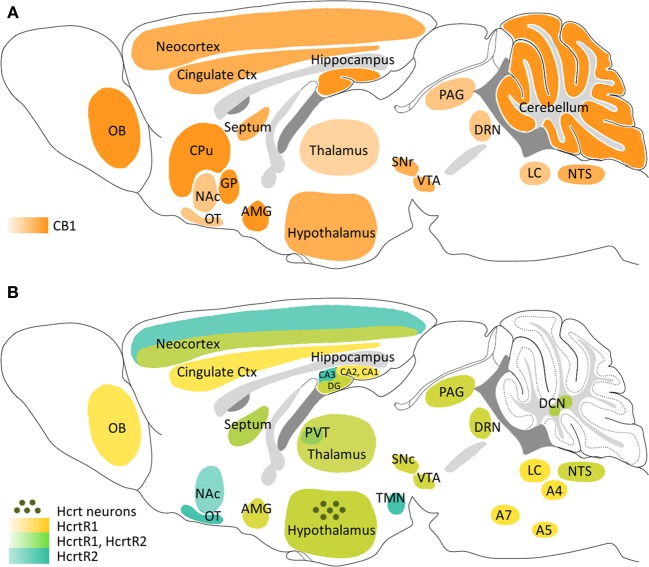
**Schematic representation of the main areas expressing CB1, HcrtR1 and HcrtR2 in the mouse brain and location of hypocretinergic neurons. (A)** CB1 receptor distribution. **(B)** HcrtR1 and HcrtR2 distribution and localization of hypocretinergic neurons. A4, A5, A7, pons cell groups; AMG, amygdala; CPu, caudate putamen; Ctx, cortex; DCN, deep cerebellar nuclei; DRN, dorsal raphe nucleus; GP, globus pallidus; LC, locus coeruleus; NAc, nucleus accumbens; NTS, nucleus of the solitary tract; OB, olfactory bulb; OT, olfactory tubercle; PAG, periaqueductal gray; PVT, paraventricular nucleus of thalamus; SNc, substantia nigra pars compacta; SNr, substantia nigra pars reticulata; TMN, tuberomammillary nucleus; VTA, ventral tegmental area.

Anatomical studies have found that CB1 and HcrtRs show an overlapping distribution in several areas of the CNS (Hervieu et al., 2001; Marcus et al., 2001; Mackie, 2005), suggesting a common role in some physiological functions (Figure 1). Thus, HcrtR1 and HcrtR2, as well as CB1, are widely expressed within the entire hypothalamus, denoting an important function of these systems in energy homeostasis and central regulation of neuroendocrine and autonomic functions (Wittmann et al., 2007; Tsujino and Sakurai, 2009). Both receptors are also found in diverse areas of the mesocorticolimbic system, such as the ventral tegmental area (VTA), the nucleus accumbens (NAc), the prefrontal cortex (PFC), the septal nuclei and the amygdaloid nuclei, supporting the regulation of natural reward and addiction processes by endocannabinoid and hypocretinergic systems (Maldonado et al., 2006; Aston-Jones et al., 2010; Plaza-Zabala et al., 2012). The presence of CB1, HcrtR1, and HcrtR2 within diverse brainstem nuclei, including the raphe nuclei, the locus coeruleus, the reticular formation and the periaqueductal gray (PAG), is in agreement with the role of these neuromodulators with the regulation of anxiety-like responses, sleep/wake cycle and nociception (Eriksson et al., 2010; Häring et al., 2012; Wilson-Poe et al., 2012). Nevertheless, the existence of cross-reactivity problems with HcrtR antibodies has hindered the precise location of these receptors (Kukkonen, 2012). Hence, although specific CB1 expression among different neuronal populations has been well characterized (Mackie, 2005), the location of HcrtRs is certainly known only at the level of brain structure since it has been confirmed by *in situ* hybridization studies (Hervieu et al., 2001). As a consequence, direct synaptic connections between CB1 and HcrtRs are not well defined. On the other hand, recent studies have detected certain multifocal expression of CB2 in the brain at levels much lower than those of CB1 receptors (Gong et al., 2006; Onaivi et al., 2006). Among these CB2 expression foci, hippocampus, amygdala and PAG are potentially the most relevant areas to the study of the cannabinoid-hypocretinergic interplay.

**Figure 2:**
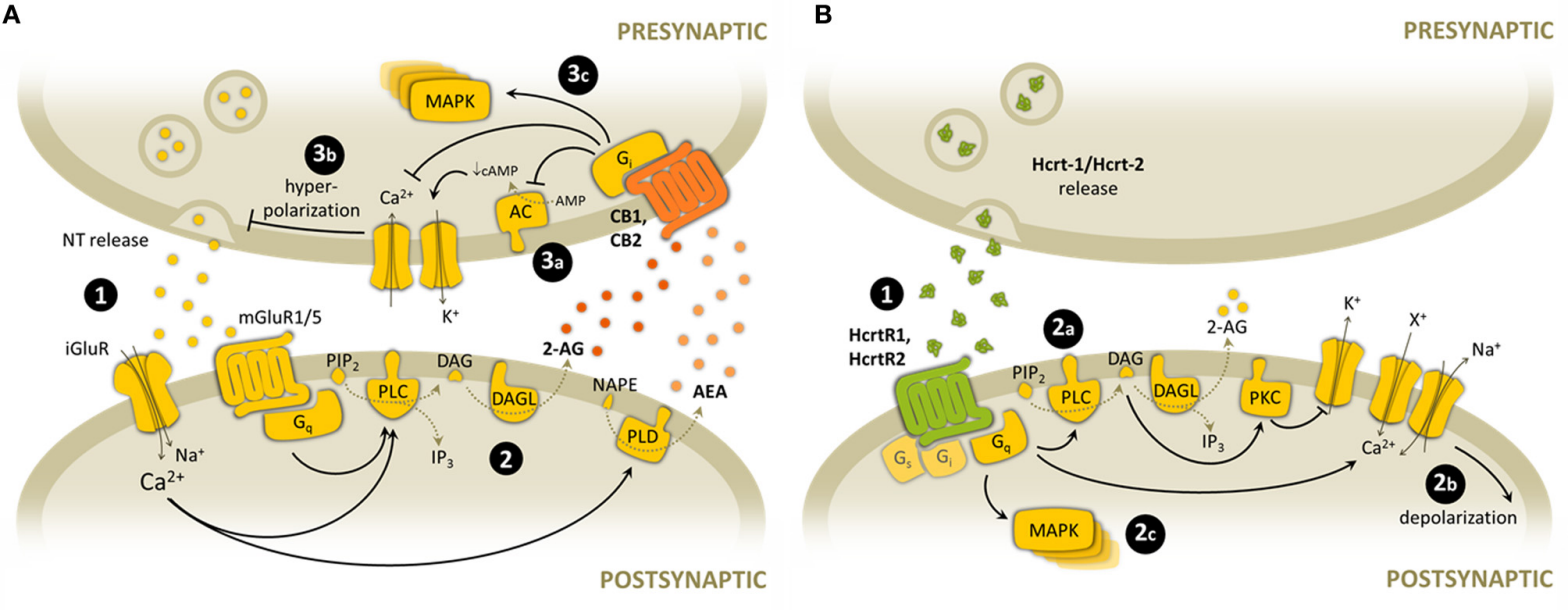
**Overview of the main synaptic signaling mechanisms of endocannabinoid and hypocretinergic systems. (A)** Endocannabinoid-mediated synaptic signaling. (1) Glutamate is released from presynaptic terminals and stimulates both ionotropic and metabotropic glutamate receptors, leading to postsynaptic depolarization through Ca^2+^ entrance and Gq-protein activation. (2) High Ca^2+^ concentration stimulates endocannabinoid synthesis through PLC and PLD. 2-AG synthesis is also mediated by Gq-protein activation. (3) Endocannabinoids are released to the synaptic cleft and activate CB1 and CB2 presynaptic receptors. Some of the main downstream consequences of CB receptor activation and subsequent Gi-protein stimulation are: (3a) inhibition of AC activity, (3b) membrane hyperpolarization after modulation of K+ and Ca2+ channels, and subsequent inhibition of NT release, (3c) activation of protein kinase cascades such as MAPK pathway. **(B)** Hypocretin-mediated synaptic signaling. (1) Hypocretins are released from presynaptic terminals and activate postsynaptic HcrtR1 and HcrtR2. (2) HcrtR stimulation is mainly associated with Gq-protein activation, but it can activate also other G-protein subtypes. Some of the main downstream consequences of HcrtR activation and subsequent Gq-protein stimulation are: (2a) activation of PLC activity, and subsequent DAG and 2-AG synthesis (2b) membrane depolarization after modulation of K+ channels, non-specific cationic channels and Na^+^/Ca^2+^ exchanger, (2c) activation of protein kinase cascades such as MAPK pathway. NT, neurotransmitter; iGluR, ionotropic glutamate receptor; mGluR, metabotropic glutamate receptor; PIP2, phosphatidylinositol bisphosphate; DAG, diacylglicerol; 2-AG, 2-arachidonoylglycerol; NAPE, N-arachidonoyl-phosphatidylethanolamine; AEA, anandamide; PLC, phospholipase C; DAGL, diacylglycerol lipase; PLD, phospholipase D; AC, adenyl cyclase; cAMP, cyclic AMP; MAPK, mitogen-activated protein kinase; Hcrt-1, hypocretin-1; Hcrt-2, hypocretin-2; PKC, protein kinase C; X^+^, unspecific cation.

CB1 and CB2, as well as HcrtR1 and HcrtR2, belong to the rhodopsin subfamily of GPCR superfamily. The cellular signals triggered upon cannabinoid receptor activation differ from those initiated following the stimulation of hypocretin receptor. However, it seems that diverse signaling pathways are common for cannabinoid and hypocretin receptors (Demuth and Molleman, 2006) (Figure 2). Both CB1 and CB2 receptors are associated with the Gi/o family of G-proteins, as most cannabinoid effects are blocked by pertussis toxin (PTX) (Howlett et al., 1986; Slipetz et al., 1995). Subsequent functional inhibition of adenylyl cyclase (AC) activity and decreased cAMP production has been observed in most tissues and cells investigated (Howlett et al., 2002). However, CB1 has been shown to stimulate AC when Gi protein is hardly available, such as under PTX treatment or sequestering by other GPCR receptor activation, indicating that CB1 may be able to couple Gs under these particular experimental conditions (Glass and Felder, 1997; Jarrahian et al., 2004). The modulation of voltage-dependent ion channels by CB1 activation is thought to underlie the cannabinoid-induced inhibition of neurotransmitter release, although it seems that CB1-independent mechanisms of ion channel modulation might also exist (Demuth and Molleman, 2006). CB1 activates inward-rectifying K^+^ (Kir) and A-type K^+^ channels, triggering the plasmatic membrane repolarization (Deadwyler et al., 1995; Vásquez et al., 2003). This was shown to be mediated by CB1 receptor-mediated reduction in cAMP levels and PKA activation (Deadwyler et al., 1995; Hampson et al., 1995). Additionally, CB1 inhibits N-, P/Q- and L-type voltage-gated Ca^2+^ channels, leading to a decrease in Ca^2+^ influx, mostly by direct Gβγ interaction with the channel (Howlett et al., 2002). CB1 and CB2 activation also leads to the phosphorylation and activation of the MAP kinase cascade (Bouaboula et al., 1995, 1997; Derkinderen et al., 2001), which regulates neuronal gene expression and synaptic plasticity. Diverse transduction pathways leading to activation of different MAP kinases (ERK1/2, JNK, ERK5, and p38) have been proposed, depending on the cell type and the stimulus. MAP kinase activation is mediated by PI3K pathway in CHO cells (Galve-Roperh et al., 2002), PC-3 cells (Sánchez et al., 2003) and astrocytoma cells (Gómez del Pulgar et al., 2000), through the protein kinase B (PKB/Akt) phosphorylation and Raf-1 activation. Some studies also suggest that decrease in cAMP levels, and consequently reduced inhibitory c-Raf phosphorylation by PKA activity, may participate in the stimulatory effects of CB1 activation on the MAP kinase pathway (Melck et al., 1999; Davis et al., 2003).

On the other hand, both hypocretin receptors are coupled to Gq proteins, which induce the activation of PLC and production of the second messengers DAG and IP3 from PIP2. This triggers the activation of PKC, which phosphorylates and modulates effector ion channels leading to Ca^2+^ entrance (van den Pol et al., 1998; Eriksson et al., 2001), as well as further IP3-mediated entry via store-operated Ca^2+^ channels (Kukkonen and Akerman, 2001; Larsson et al., 2005). In addition, membrane depolarization is facilitated by activation of Na^+^/Ca^2+^ exchanger (Burdakov et al., 2003), increase of non-selective cation channel conductances (Liu et al., 2002; Yang and Ferguson, 2002; Murai and Akaike, 2005) and/or blockade of Kir channels (Hwang et al., 2001; Yang and Ferguson, 2003; Ishibashi et al., 2005). It remains to be further elucidated by using selective antagonists the identification of the receptor subtype mediating these effects. Additionally, some studies of lipid signaling pathways activated by HcrtR1-expressing CHO cells have also revealed coupling to PLD and PLA2 (Turunen et al., 2012). Besides, stimulation of both hypocretin receptors has been suggested to modulate AC activity by coupling other G-proteins, such as Gs-protein as shown by AC activation and cAMP production in neurons (Gorojankina et al., 2007) and astrocytes (Woldan-Tambor et al., 2011), or Gi-protein as observed by hypocretin-1 inhibition of AC via Gi-coupling (Holmqvist et al., 2005; Urbańska et al., 2012). Similar to cannabinoids, hypocretin signaling also activates the MAP kinase pathway. Thus, HcrtR1 challenge leads to ERK1/2 and p38 kinase phosphorylation (Ammoun et al., 2006a). Downstream effectors contributing to ERK1/2 activation after HcrtR1 stimulation include at least Ca^2+^ influx, PLC/PKC, Ras, Src, and PI3K (Ammoun et al., 2006b). Similar results have been recorded in an HcrtR2 expression system (Tang et al., 2008). Thus, cannabinoid and hypocretinergic signaling differ in their modulation of ion channel currents and AC activity, while they converge in the activation of the MAP kinase pathway.

## Molecular interactions between CB1 and HcrtR1

Direct CB1-HcrtR1 interaction was first proposed in 2003 (Hilairet et al., 2003). Indeed, a 100-fold increase in the potency of hypocretin-1 to activate the ERK signaling was observed when CB1 and HcrtR1 were co-expressed in CHO cells. This effect required a functional CB1 receptor as evidenced by the blockade of hypocretin response by the CB1 antagonist rimonabant, and was blocked by PTX, suggesting a Gi-mediated potentiation. Based on electron microscopy colocalization, the authors inferred the formation of heteromeric complexes by HcrtR1 and CB1 that might explain the enhancement in hypocretin-induced ERK signaling (Hilairet et al., 2003). Importantly, in these colocalization studies specificity problems with anti-HcrtR1 antibodies were avoided by tagging the N-terminus of HcrtR1 with the c-Myc epitope, monitoring its expression using mouse monoclonal anti-Myc antibodies. The possible existence of CB1-HcrtR1 heteromerization has been further assessed by co-expressing these GPCRs in HEK293 cells (Ellis et al., 2006). In this study, rimonabant caused a decrease in the potency of hypocretin-1 to activate the MAP kinases ERK1/2 in cells co-expressing both receptors. Similarly, the HcrtR1 antagonist SB674042 reduced in these cells the potency of the CB1 agonist WIN55,212-2 to phosphorylate ERK1/2. Additionally, co-expression of CB1 and HcrtR1 resulted in coordinated trafficking of these GPCRs. Indeed, following inducible expression in HEK293 cells, HcrtR1 was mainly located in the cell surface, while CB1 constitutive expression resulted in a distribution pattern in intracellular vesicles consistent with spontaneous, agonist-independent internalization. When both receptors were co-expressed, HcrtR1 appeared to be recycled in intracellular vesicles, adopting the location of CB1 inherent to this model. When treated with rimonabant or with SB674042, both CB1 and HcrtR1 were re-localized at the cell surface. The possible direct protein-protein interaction between CB1 and HcrtR1 deduced from these data was tested by performing single cell fluorescence resonance energy transfer (FRET) imaging studies, which confirmed that CB1 and HcrtR1 were close enough to form veritable heteromers (Ellis et al., 2006). Recently, the same group has demonstrated further evidence of such heteromerization by covalently labeling the extracellular domains of CB1 and HcrtR1 with SNAP-tag® and CLIP-tag™ labeling systems, which consist in two polypeptides that can be fused to a protein of interest and further covalently tagged with a suitable ligand (i.e., a fluorescent dye), allowing a reliable monitorization of these heteromers at the cell surface (Ward et al., 2011a,b). In this study, a higher potency of hypocretin-1 to regulate CB1-HcrtR1 heteromer compared with the HcrtR1-HcrtR1 homomer was reported (Ward et al., 2011b). These data provide unambiguous identification of CB1-HcrtR1 heteromerization, which has a substantial functional impact.

Besides the heteromerization, an additional mechanism has been proposed to explain the increase in the potency of hypocretin-1 to activate the ERK pathway in the presence of CB1 (Jäntti et al., 2013; Kukkonen and Leonard, 2013). Recent studies report that HcrtR1-expressing CHO cells may release 2-AG in response to hypocretin-1 stimulation. In these cells, the activation of PLC is responsible for DAG production, which in turn is used by diacylglycerol lipase (DAGL) as a substrate for 2-AG production (Turunen et al., 2012). Taking into account that both HcrtR1 and CB1 activate ERK upon ligand binding (Bouaboula et al., 1995; Ammoun et al., 2006a), it is possible that 2-AG-mediated stimulation of CB1 could contribute to increase the potency of hypocretin-1 signaling in the CHO cell expression system. In addition, recent evidence supports that endocannabinoids may act in an auto- or paracrine manner, and the influence of endogenously produced endocannabinoids when introducing Gq-coupled receptors to the expression system cannot be discarded (Howlett et al., 2011). Indeed, it has been demonstrated that HcrtR1 stimulation elevates 2-AG in biologically relevant quantities, activating CB1 receptors in nearby cells (Turunen et al., 2012). Importantly, this hypocretin-induced endocannabinoid release might shed light on the mechanisms by which hypocretins mediate synaptic inhibition in certain conditions.

## Functional interaction between cannabinoids and hypocretins: emerging studies

Despite anatomical, biochemical and pharmacological evidence supporting the possible existence of a link between cannabinoids and hypocretins, few studies have directly evaluated this cross-talk at the functional level (Table 1). Current research suggests their mutual involvement in the regulation of several physiological responses including appetite, reward, sleep/wake cycle and nociception.

**Table 1:** **Studies investigating the interaction between endocannabinoid and hypocretinergic systems**.

**Functional interaction**	**Tools**		**Techniques**	**Main result**	**References**
Energy balance	*in vivo* (rat)	Hypocretin-1	Food intake monitoring	Subeffective systemic rimonabant attenuates food intake induced by central hypocretin-1	Crespo et al., 2008
		CB1 antagonist			
	Rat tissue	CB1 antagonist	LH Immunofluorescence	Rimonabant administration induces Fos expression in hypocretin, MCH, MSH and CART neurons, but does not affect hypocretin mRNA or protein levels	Verty et al., 2009
			qRT-PCR		
			Western Blot		
	*ex vivo* (mouse)	Obese *ob/ob* mice	Confocal, electron microscopy	In obesity, hypocretinergic neurons overexpress DAGL and receive predominantly inhibitory, instead of excitatory, CB1-expressing inputs. These alterations are reversed by leptin administration	Cristino et al., 2013
		High-fat diet	LH electrophysiology		
		CB agonist	LH immunofluorescence		
		Leptin			
		mTOR inhibitor			
Reward and Cannabis dependence	*in vivo* (rat)	Cholinergic agonist	Conditioned place preference	CPP induced by LH-chemical stimulation requires HcrtR1 and CB1 receptor signaling in the VTA	Taslimi et al., 2011
		HcrtR1 antagonist			
		CB1 antagonist			
	*in vivo* (mouse)	HcrtR1 knockout mice	Drug self-administration	Rewarding properties of cannabinoids are modulated by HcrtR1 and activate LH hypocretinergic neurons. HcrtR1 regulates THC-induced dopamine release in Nac	Flores et al., 2013
		CB agonists	LH immunofluorescence		
		HcrtR1 and HcrtR2 antagonists	*In vivo* microdyalisis		
	*in vivo* (human)	–	Peripheral blood gene expression and promoter metylation study	THC-smokers show decreased hypocretin expression when compared to cigarette-smokers	Rotter et al., 2012
Antinociception	*ex vivo* and *in vivo* (rat)	Hypocretin-1	PAG electrophysiology	Hypocretin-1 inhibits GABA release to PAG neurons through retrograde 2-AG signaling, leading to increased PAG activity.	Ho et al., 2011
		HcrtR1 and HcrtR2 antagonists	PAG Immunofluorescence	Antinociceptive responses induced by intra-PAG administration of hypocretin-1 are mediated by HcrtR1 and CB1 receptors	
		CB1 agonist and antagonist	Hot plate test		
		PLC and DAGL inhibitors			
Sleep/wake cycle	*in vivo* (rat)	CB1 agonist and antagonist	EEG and EMG monitoring	Intra-LH administration of 2-AG increases REM sleep and cFos expression in MCH neurons, but does not affect cFos expression in hypocretinergic neurons	Pérez-Morales et al., 2013
			LH immunofluorescence		
	*ex vivo* (rat)	Hypocretin-2	DRN electrophysiology	Hypocretin-2 inhibits glutamate release to DRN serotonergic neurons through retrograde 2-AG signaling	Haj-Dahmane and Shen, 2005
		CB1 agonist and antagonist			
		PLC and DAGL inhibitors			
Cellular and molecular interaction	*ex vivo* (mouse)	CB agonist ad antagonist	LH electrophysiology	Cannabinoids reduce activity of hypocretin neurons by presynaptic attenuation of glutamate release and excite MCH neurons by presynaptic inhibition of GABA release	Huang et al., 2007
		GABAa antagonist	Immunocytochemistry		
		iGluR antagonist			
	*in vitro* (cell culture)	Hypocretin-1	Confocal, electron microscopy	CB1-HcrtR1 coexpression potentiates activation of the MAPK pathway induced by hypocretin-1	Hilairet et al., 2003
		CB1 antagonist	Intracellular signaling assays		
	*in vitro* (cell culture)	Hypocretin-1	Redistribution assays	CB1-HcrtR1 heteromerization results in coordinated alteration of their cellular localization and downstream signaling	Ellis et al., 2006
		CB1 agonist and antagonist	Epifluorescence microscopy		
		HcrtR1 antagonist	FRET imaging		
	*in vitro* (cell culture)	CB1 agonist and antagonist	Co-immunoprecipitation	Heteromultimerization of CB1-HcrtR1 is confirmed by co-immunoprecipitation and SNAP/CLIP tagging.	Ward et al., 2011a,b
		HcrtR1 antagonist	SNAP and CLIP tagging	Modulation of receptor internalization and MAPK pathway activation is also reproduced	
			FRET imaging		
			Intracellular signaling assays		
	*in vitro* (cell culture)	Hypocretin-1 and -2	Intracellular signaling assays	HcrtR1 stimulation by hypocretin-1 activates PLA2 and DAGL cascades with subsequent release of AA and 2-AG, which acts as paracrine messenger through CB1	Turunen et al., 2012
		CB1 agonist and antagonist			
		DAGL and MAGL inhibitors			
		PLC, PLD and PLA inhibitors			
		HcrtR1 antagonist			
	*in vitro* (cell culture)	Hypocretin-1 and -2	Intracellular signaling assays	Release of 2-AG induced by hypocretin-1 stimulates ERK activity in neighboring CB1-expressing cells. HcrtR1-mediated ERK activity is potentiated in cells coexpressing CB1-HcrtR1	Jäntti et al., 2013
		CB1 antagonist			
		HcrtR1 antagonist DAGL and MEK inhibitors			

*Abbreviations: IF, immunofluorescence; EPS, electrophysiology; drug self admin, drug self-administration; EF microscopy, epifluorescence microscopy; co-IP, co-immunoprecipitation*.

### Appetite and energy balance

The regulation of energy balance is determined by the control of food intake and energy expenditure. The so-called homeostatic control of energy balance is exerted in response to variations in the nutritional status and energy stores and is autonomic or involuntary, whereas the non-homeostatic control has a cognitive component strongly influenced by the hedonic aspects of eating (Saper et al., 2002; Berthoud, 2007) (see section Regulation of the brain rewarding system). Interestingly, endocannabinoid and hypocretinergic systems appear to be involved in both processes. Recently, the LH has been suggested to constitute a bridge between homeostatic and non-homeostatic brain areas involved in energy balance regulation. Indeed, this region connects the hypothalamic regulators of energy balance [e.g., the arcuate nucleus (Arc) and the paraventricular nucleus (PVN)], to the NAc and the VTA, two key parts of the brain reward system (Berthoud, 2007; Richard et al., 2009).

Endocannabinoids, as well as systemic administration of cannabinoid agonists, stimulate food intake (Williams et al., 1998; Williams and Kirkham, 1999). These effects are mediated by CB1 receptor. Indeed, rimonabant reduces the consumption of standard food in food-deprived animals (Colombo et al., 1998), and CB1-deficient mice consume less food than wild-type littermates and are resistant to diet-induced obesity (Di Marzo et al., 2001; Cota et al., 2003). Accordingly, fasting increases levels of anandamide and 2-AG in the limbic forebrain and, to a lesser extent, of 2-AG in the hypothalamus, whereas feeding declines endocannabinoid levels in these areas (Kirkham et al., 2002). Likewise, central administration of hypocretin-1 or hypocretin-2 stimulates food consumption, whereas systemic administration of the HcrtR1 antagonist SB334867 reduces feeding (Sakurai et al., 1998; Haynes et al., 2000; Shiraishi et al., 2000). Furthermore, preprohypocretin mRNA is upregulated following fasting (Sakurai et al., 1998) as well as in obese mice during food restriction (Yamanaka et al., 2003). Interestingly, pretreatment with a non-anorectic dose of rimonabant blocks orexigenic actions of hypocretin-1 administered by intracerebroventricular route (icv) in pre-fed rats, suggesting that hypocretin-1 exerts its orexigenic action through CB1 receptor activation (Crespo et al., 2008). However, the increase induced by hypocretin-1 in food intake correlates with an increase in locomotion and wakefulness (Yamanaka et al., 1999; Crespo et al., 2008), leading to the hypothesis that the primary function of this system is promoting arousal in response to food deprivation, which would facilitate the food consumption (Yamanaka et al., 2003; Cason et al., 2010).

**Figure 3:**
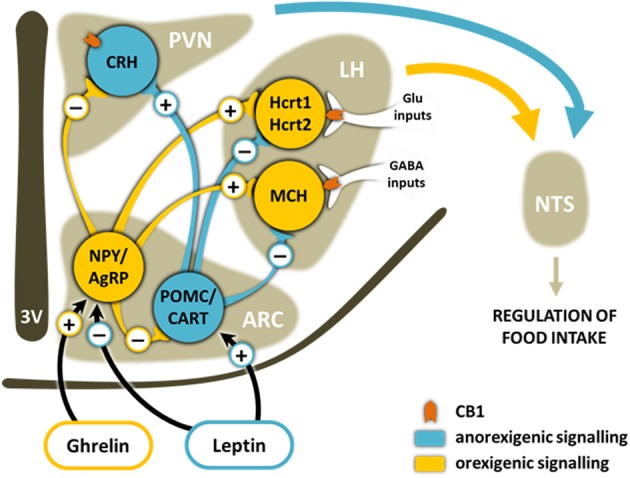
**Schematic representation of the main brain pathways involved in the homeostatic control of food intake**. Ghrelin released during fasting from stomach and leptin from adipose tissue, among other mediators, bind to receptors on orexigenic and/or anorexigenic neurons in the ARC of the hypothalamus. This induces the release of either the orexigenic neuropeptides NPY and AgRP or the anorexigenic neuropeptides CART and the POMC-derived peptide α-MSH. These neuropeptides from the ARC travel along axons to secondary neurons in other areas of the hypothalamus such as the PVN and the LH. The ultimate effects of these signaling cascades are changes in the sensation of hunger and satiety in the NTS. Hypocretinergic and MCH neurons are modulated differently by inhibitory or excitatory CB1-expressing inputs. ARC, arcuate nucleus; PVN, paraventricular nucleus; LH, lateral hypothalamus; NTS, nucleus of the tractus solitarius; 3V, third ventricle; NPY, neuropeptide Y; AgRP, Agouti-related peptide; CART, cocaine- and amphetamine-regulated transcript; POMC, pro-opiomelanocortin; MCH, melanin-concentrating hormone; CRH, corticotropin-releasing hormone; Hcrt1, hypocretin-1; Hcrt2, hypocretin-2.

One of the main hypothalamic regulators of appetite is the Arc-PVN axis (Girault et al., 2012) (Figure 3). Circulating levels of leptin, produced by adipocytes in proportion to the adipose mass, inhibit neurons in the Arc that co-express the orexigenic neurotransmitters neuropeptide Y (NPY) and agouti-related peptide (AgRP), whereas they activate the anorexic pro-opiomelanocortin (POMC) neurons that co-express cocaine-amphetamine-related transcripts (CART). Grehlin, released during fasting, produces the opposite effect on these neurons. NPY/AgRP and POMC/CART neurons convey their information to second-order neurons in the PVN and LH, such as the corticotrophin-releasing hormone (CRH), the melanin-concentrating hormone (MCH) and hypocretin neurons (Elias et al., 1998). Emerging evidence suggests that NPY and hypocretin neurons have reciprocal excitatory connections. Thus, reduced plasma glucose and leptin and increased grehlin levels induce fasting-related arousal by causing an activation of NPY neurons finally increasing the firing of hypocretin neurons. Additionally, it seems that increased hypocretinergic activity during sleep deprivation may activate NPY neurons resulting in hyperphagia independent from peripheral endocrine and metabolic signaling (Yamanaka et al., 2000).

CB1 receptors colocalize with CART, MCH and hypocretin neurons (Cota et al., 2003). Acute administration of rimonabant induces c-fos in all these neuronal populations including hypocretinergic cells, increases CART and decreases NPY expression, consistent with its anorexic effect. However, the CB1 antagonist has no effect in hypocretin expression suggesting that hypocretins are not likely to be the main mediators of cannabinoid hypothalamic orexigenic effects (Verty et al., 2009). An interesting electrophysiological study in mouse reveals that the cannabinoid agonist WIN55-212,2 depolarizes MCH cells increasing spike frequency while reducing spontaneous firing of hypocretin cells (Huang et al., 2007). CB1-mediated depolarization of MCH cells was a consequence of cannabinoid action on axons arising from LH local inhibitory cells, resulting in reduced synaptic GABA release on MCH neurons. On the contrary, CB1 agonists hyperpolarized hypocretin cells by presynaptic attenuation of glutamate release (Huang et al., 2007). These results are in line with the idea that some of the orexigenic actions of cannabinoids could be explained by their synaptic effects on MCH neurons, which undoubtedly regulate energy balance (Pissios et al., 2006), and are not mediated by hypocretin neurons at least in physiological conditions. However, a recent study shows that the balance between CB1-expressing glutamatergic and GABAergic inputs to hypocretin neurons is altered in obesity (Cristino et al., 2013). In leptin-knockout (ob/ob) obese mice and in diet-induced obese mice, hypocretin neurons appear to receive predominantly inhibitory instead of excitatory CB1-expressing inputs (Cristino et al., 2013). In addition, hypocretin neurons overexpress the main enzyme that synthesizes 2-AG, DAGLα, in these obesity models, in line with previous results reporting elevated hypothalamic levels of endocannabinoids in ob/ob mice and obese Zucker rats (Di Marzo et al., 2001). These alterations could result in a retrograde inhibition of these inhibitory CB1-expressing axon terminals, leading to disinhibition of hypocretinergic neurons and enhancing hypocretin innervation of target brain areas. This remodeling could be a consequence of leptin signaling impairment, since it was reversed by the systemic administration of leptin (Cristino et al., 2013). Therefore, in these pathological conditions, hypocretins could exacerbate obesity as a result of an increased hypocretin release in the hypothalamus, leading to hyperphagia and sleep disorders (Alpár and Harkany, 2013).

Another relevant integrative region in the control of appetite is the nucleus of the solitary tract (NTS) in the hindbrain, which regulates individual meal sizes and the intervals between meals (Valassi et al., 2008). Peripheral satiety signals are relayed to the NTS via vagal afferent neurons, whose cell bodies lie in the nodose ganglia. It has been recently reported that fasting-induced increase in CB1 immunoreactivity has been observed under standard food regime in the nodose ganglia (Cluny et al., 2013). In contrast, OX-1R immunoreactivity is modified by fasting only in rats exposed to high-fat diet, denoting a differential regulation of these neurotransmitter systems in this structure (Cluny et al., 2013). On the other hand, hypocretin-1 depolarizes NTS neurons through regulation of non-selective cationic and K+ conductances in a PKC-dependent manner (Yang et al., 2003; Yang and Ferguson, 2003). It has ben also reported that CB1 receptor activation modulates NTS neuronal activity (Seagard et al., 2005; Endoh, 2006). However, it remains to be investigated whether these neurochemical effects in nodose ganglia and NTS are relevant in the control of feeding behavior, and if there exists an interplay between endocannabinoid and hypocretinergic mechanisms.

Endocannabinoid and hypocretinergic systems seem to play antagonistic roles in the peripheral control of energy balance. The reduction of body weight and fat mass exerted by CB1 antagonists in diet-induced obesity models is partially due to the counteraction of a peripheral stimulation of lipogenesis by endocannabinoids (Di Marzo and Matias, 2005). Moreover, chronic CB1 blockade improves peripheral metabolic parameters of obesity, including a reduction in plasma levels of insulin and leptin. In contrast, mice overproducing the hypocretin peptides exhibit resistance to high-fat diet-induced obesity at least in part by promoting energy expenditure (Tsuneki et al., 2010). These antiobesity metabolic effects have been demonstrated to be mediated by HcrtR2, which has been observed to improve leptin sensitivity (Funato et al., 2009). However, no studies have been yet published assessing whether endocannabinoid and hypocretinergic systems show a cross-modulation in their peripheral control of metabolic rates.

### Regulation of the brain reward system

Endocannabinoid and hypocretinergic systems are also involved in the regulation of the mesocorticolimbic rewarding system, a circuit responsible for the pleasurable feelings associated with natural rewards and the consumption of drugs of abuse. The major components of this reward circuit are the VTA, which contains the dopaminergic cell bodies, and its target areas, including the NAc, amygdala, frontal and limbic cortices (Wise, 2004). CB1 and HcrtRs receptors are abundant in the brain reward circuitry and participate in the rewarding properties of natural rewards and also in those induced by different drugs of abuse (Maldonado et al., 2006). Acting as a retrograde messenger, endocannabinoids modulate the glutamatergic excitatory and GABAergic inhibitory synaptic inputs into the dopaminergic neurons of the VTA and the glutamate transmission in the NAc. Thus, the activation of CB1 receptors present on axon terminals of GABAergic neurons in the VTA inhibits GABA transmission, removing this inhibitory input on dopaminergic neurons (Riegel and Lupica, 2004). Glutamate synaptic transmission in the VTA and NAc, mainly from neurons of the PFC, is similarly modulated by the activation of CB1 receptors (Melis et al., 2004). The final effect of endocannabinoids on the modulation of dopaminergic activity, which depends on the functional balance between these GABAergic and glutamatergic inputs, is predominantly excitatory (Maldonado et al., 2006). On the other hand, hypocretins regulate reward seeking also by modulating VTA dopaminergic transmission. In agreement, intra-VTA infusion of hypocretin-1 and -2 increased dopamine release in NAc and PFC as measured by microdialysis or voltammetry (Vittoz et al., 2008; España et al., 2011). Hypocretins elicit their influence on VTA dopamine cell firing not only via direct depolarization of dopamine neurons (Korotkova et al., 2003), but also interacting with other neurotransmitters within the VTA, such as glutamate (Borgland et al., 2006). Thus, intra-VTA infusion of hypocretin-1 increased both glutamate and dopamine release, which was attenuated by the AMPA/NMDA antagonist kynurenic acid, suggesting that hypocretin has a profound influence on dopamine neurons by affecting glutamatergic activity (Wang et al., 2009). Hypocretin-1 enhanced glutamatergic synaptic strength on dopamine neurons in VTA slices (Borgland et al., 2006). In accordance, the control of this limbic structure by the PFC projections was improved by hypocretin-1 in rats (Mahler et al., 2013).

This considerable modulation of the reward circuit by endocannabinoid and hypocretinergic systems reveal their important role in the non-homeostatic control of food intake. CB1 receptor antagonists have been reported to reduce the conditioned place preference (CPP) for food (Chaperon et al., 1998), and the motivation for food in a progressive ratio schedule of food self-administration in rats (Gallate and McGregor, 1999). According to this, null mutant CB1 mice show a reduced motivation to work for food compared to wild-type littermates (Sanchis-Segura et al., 2004). CB1 receptor blockade also decreases the reinforcing properties of chocolate and sweets (Maccioni et al., 2008). In addition, intra-NAc injections of anandamide enhance the reward associated with sweets (Mahler et al., 2007). Similarly, several data support a role for hypocretins in food-seeking and taking. Thus, chronic administration of the HcrtR1 antagonist SB334867 altered standard food self-administration in food-restricted mice (Sharf et al., 2010), although this effect was not observed in rats after acute administration of the HcrtR1 antagonist (Borgland et al., 2009). Indeed, it seems that hypocretinergic control of food-related reward is more relevant when it involves particularly palatable foods (Mahler et al., 2012). In agreement, SB334867 reduces both motivational and primary reinforcing effects in rats trained to self-administer high-fat food, both under food-restriction or satiation (Nair et al., 2008; Choi et al., 2010). So far, no studies have investigated if endocannabinoid and hypocretinergic systems have common mechanisms in the modulation of natural reward. However, it seems that both neurotransmitter systems regulate food intake especially when particularly palatable/or salient food is involved or higher effort is required to obtain this natural reinforcer.

Similarly, the addictive properties of several drugs of abuse are modulated by hypocretinergic and endocannabinoid systems. Hypocretin transmission regulates the primary reinforcing effects of opioids (Narita et al., 2006; Smith and Aston-Jones, 2012), nicotine (Hollander et al., 2008; LeSage et al., 2010) and alcohol (Lawrence et al., 2006; Moorman and Aston-Jones, 2009). However, the involvement of the hypocretin system in the rewarding properties of psychostimulants seems to be relevant only under conditions that require a high effort to obtain the drug (Boutrel et al., 2005; España et al., 2011). This distinct regulation could be due to differences among the mechanism of action by which these drugs of abuse alter the mesolimbic function (Plaza-Zabala et al., 2012). Opioids, nicotine and alcohol increase extracellular levels of dopamine in the NAc by enhancing dopaminergic firing rates in the VTA, whereas psychostimulants directly inhibit dopamine uptake in the NAc (Di Chiara et al., 2004). Thus, rewarding effects of drugs of abuse that depend on increased VTA dopaminergic activity may require hypocretinergic transmission, as the VTA appears to be an essential site of action for hypocretins to modulate these effects. In contrast, the mechanism of action of psychostimulants avoids this critical site of action of hypocretins. Intriguingly, a similar phenomenon occurs with the modulatory role of the endocannabinoid system on the primary rewarding effects of drugs of abuse. Thus, opioid (Navarro et al., 2001, 2004), cannabinoid (Maldonado et al., 2006), nicotine (Castañé et al., 2002; Cohen et al., 2002) and alcohol (Hungund et al., 2003; Wang et al., 2003) reinforcement depend on endocannabinoid signaling in the VTA, but primary rewarding effects of psychostimulants remain unaffected in the absence of CB1 receptors (Martin et al., 2000; Soria et al., 2005). However, the endocannabinoid system is important for maintaining psychostimulants seeking behavior when higher effort is required to obtain the drug, probably by the modulation of other mechanisms independent from release of dopamine in the NAc (Soria et al., 2005). It has been recently reported that CPP induced by chemical stimulation of the LH with the cholinergic agonist carbachol is regulated by HcrtR1 activation in the VTA. Thus, unilateral intra-VTA administration of the HcrtR1 antagonist SB334867 dose-dependently inhibited this behavioral response (Taslimi et al., 2011). Interestingly, intra-VTA administration of rimonabant also decreased CPP induced by LH-stimulation in a dose-dependent manner. Co-administration of effective doses of both HcrtR1 and CB1 antagonists into the VTA reduced CPP in a non-additive manner, suggesting that these receptors regulate this effect by a common mechanism (Taslimi et al., 2011). Nevertheless, future experiments showing the specific location of HcrtR1 and CB1 receptors within the VTA neurons will be necessary to better understand the interaction between the endocannabinoid and hypocretin systems in the regulation of the reward circuit.

A relevant but almost unexplored aspect of hypocretin-cannabinoid interplay is the role of the hypocretinergic system in the addictive properties of cannabinoids, whose recreational use has progressively increased in developed countries in the last decade (Murray et al., 2007). Therefore, the identification of new therapeutic targets to improve treatment outcomes for cannabis dependence is imperative considering that no effective pharmacotherapeutic approaches for this disorder are currently available. Although operant responding for self-infused THC has not been consistently reported in rodents, intravenous self-administration of the synthetic cannabinoid WIN55,212-2 has been observed in rats and mice (Fattore et al., 2001; Mendizábal et al., 2006). The reinforcing properties of cannabinoids have been related to their capability to enhance dopamine extracellular levels in the NAc shell (Fadda et al., 2006; Lecca et al., 2006), although other neurochemical systems have also been involved in cannabinoid reward, such as opioid, noradrenaline, serotonine, acetylcholine and adenosine systems (Maldonado et al., 2011).

Recently, the hypocretinergic system has also been reported to contribute to cannabinoid-induced reward. Indeed, genetic deletion or pharmacological blockade of HcrtR1 reduced the reinforcing effects of WIN55,212-2, as revealed by impaired intravenous self-administration of this synthetic cannabinoid in mice (Flores et al., 2013). In contrast, the HcrtR2 antagonist TCSOX229 had no effect in these behavioral responses. The enhancement in dopamine extracellular levels in the nucleus accumbens induced by THC was also blocked in mice lacking the HcrtR1, suggesting that cannabinoids require hypocretinergic transmission to induce dopamine release in the NAc. Moreover, contingent WIN55,212-2 self-administration, but not passive exposure to the cannabinoid, increased the percentage of hypocretin neurons expressing FosB/ΔFosB in the LH, revealing that this activation was mainly due to operant seeking for the reinforcing effects of this drug and not to its pharmacological responses (Flores et al., 2013). Cannabinoid-induced activation of hypocretin neurons reported in this study differs from previous electrophysiological data supporting that cannabinoids inhibit hypocretin neurons by CB1-mediated attenuation of glutamate release in these cells (Huang et al., 2007), but possible clarification of this divergence has recently emerged. As previously mentioned, a switch in CB1-mediated control of GABAergic and glutamatergic inputs is observed in obese mice (Cristino et al., 2013). It is thus reasonable that if this synaptic remodeling takes place in determined pathological conditions, such as obesity, the development of drug addiction might entail similar consequences by different mechanisms. However, this possibility still remains to be elucidated. Other recent evidence supports the relationship between hypocretins and cannabis dependence. It has been reported that hypocretin-1 expression in peripheral blood cells is modified in cannabis-dependent smokers when compared to nicotine-dependent smokers and non-smokers (Rotter et al., 2012). However, these data provide poor functional information, as peripheral hypocretin mRNA levels do not necessarily reflect the situation in the CNS. Moreover, it is likely that these differences are more related to peripheral actions of THC and not to the central effects involved in the development of dependence.

### Nociception

Analgesia is one of the main therapeutic targets of cannabinoids. CB1 receptors are highly expressed in pain transmission and modulation regions such as PAG, rostroventral medulla (RVM), spinal cord and primary afferent fibers (Hohmann and Suplita, 2006). Consistent with this anatomic location, several animal studies demonstrated that both endogenous and exogenous cannabinoids produce antinociceptive effects in different animal models mainly through the activation of CB1 receptors (Martin et al., 1993; Herzberg et al., 1997; Dogrul et al., 2002). Moreover, CB2 receptors have been reported to contribute to antinociception in some chronic pain models (Racz et al., 2008; La Porta et al., 2013). Cannabinoid-mediated antinociception takes places at peripheral, spinal and supraspinal levels. One of the best characterized mechanisms of pain modulation is the descending inhibitory pathway. This descending modulatory mechanism originates in the PAG, which activates neurons in the RVM, the main relay station between the PAG and spinal cord. RVM neurons send inhibitory projections to the dorsal horn of the spinal cord via dorsolateral funiculus (DLF) and modulate pain perception at the spinal level (Ren and Dubner, 2008). The demonstration that cannabinoid antinociceptive effects are diminished following surgical DLF lesion provides evidence that descending pain modulatory pathways play a crucial role in these responses (Lichtman and Martin, 1991). Thus, microinjection of the cannabinoid agonists HU210 and WIN55,212-2 into the PAG elicits antinociception mediated by CB1 receptor activation (Lichtman et al., 1996; Finn et al., 2003). This effect is the result of disinhibition of GABAergic output neurons in the PAG that leads to activation of descending inhibitory pain pathways (Vaughan et al., 2000). Similarly, local injection of CB1 agonists into RVM had antinociceptive effects due to presynaptic inhibition of GABAergic tone (Vaughan et al., 2000). Moreover, anandamide and 2-AG levels were increased in RVM in some models of chronic pain, presumably as an adaptive mechanism to counteract pain transmission (Petrosino et al., 2007).

Antinociceptive effects of hypocretins have been shown in several pain models (Chiou et al., 2010). Thus, hypocretin-1 administration by central or systemic route reduces the nociceptive responses in mice in response to thermal, mechanical and chemical stimuli (Mobarakeh et al., 2005). Hypocretin-2 has also been reported to induce antinociceptive effects in some pain models, but with lower potency than hypocretin-1 (Mobarakeh et al., 2005). Hypocretin-induced antinociception seems to be mainly mediated by HcrtR1, as revealed by using selective antagonists (Bingham et al., 2001). Hypocretin-containing fibers and HcrtRs are densely distributed in several regions of the CNS involved in the regulation of pain, including the PAG and spinal dorsal horn (Peyron et al., 1998; Marcus et al., 2001). Like cannabinoids, hypocretins appear to modulate pain perception at both spinal and supraspinal levels, but the mechanism of action remains unclear. The midbrain PAG is one of the possible supraspinal sites of hypocretin antinociception. Interestingly, PAG c-fos expression was elevated following central hypocretin administration (Date et al., 1999). In agreement, microinjection of hypocretin-1 into the PAG reduced hot-plate nociceptive responses in mice (Lee and Chiou, 2009) and formalin-induced nociceptive behaviors in rats (Yamamoto et al., 2002). A recent study in PAG slices revealed that hypocretin-1 induces inhibition of GABAergic transmission, producing an overall excitatory effect on evoked postsynaptic potentials and hence increasing PAG neuronal activity (Ho et al., 2011). This effect was blocked by the HcrtR1 antagonist SB334867, but not by the HcrtR2 antagonist TCSOX229. Moreover, the CB1 antagonist AM251 reversed the effect of hypocretin-1. Administration of U73122 and tetrahydrolipstatin, inhibitors of PLC and DAGL respectively, blocked the inhibition of GABAergic tone induced by hypocretin-1, while the inhibitor of the enzymatic degradation of 2-AG, URB602, enhanced this hypocretin effect. Therefore, hypocretin-1 may produce antinociception in part by activating postsynaptic HcrtR1 receptors and stimulating synthesis of 2-AG through a PLC–DAGL enzymatic cascade, culminating in retrograde inhibition of GABA release in the PAG. The *in vivo* existence of such analgesic mechanism was confirmed by systemic administration of SB334867 and AM251 after intra-PAG microinjection of hypocretin-1, which almost fully reversed the antinociceptive responses in the hot-plate test in rats (Ho et al., 2011). Importantly, this 2-AG-mediated antinociception induced by hypocretin signaling may contribute to stress-induced analgesia, since hypocretins have been reported to modulate this response to stress (Watanabe et al., 2005; Xie et al., 2008) and endocannabinoids within the PAG are also believed to be involved in this effect (Hohmann and Suplita, 2006). Therefore, under stressful conditions, activation of HcrtR1 may lead to PAG stimulation and produce analgesia through 2-AG via the CB1–PLC–DAGL cascade.

Both endocannabinoid and hypocretinergic systems exert also antinociceptive effects at the spinal (Drew et al., 2000; Grudt et al., 2002) and peripheral levels (Millns et al., 2001; Yan et al., 2008), but no data are available regarding a possible contribution of endocannabinoids in spinal or peripheral hypocretin-induced analgesia. Moreover, the possible modulation of cannabinoid-induced antinociception by hypocretins has not been studied yet.

### Sleep/wake cycle

Several data indicates that the hypocretinergic system is involved in the regulation of sleep-wake cycle. Thus, hypocretin neurons fire at maximal rate in wakefulness and remain silent in rapid eye movement (REM) sleep (Lee et al., 2005). Likewise, pharmacological stimulation of the hypocretinergic system increases wakefulness and reduces REM sleep (Akanmu and Honda, 2005). Accordingly, the dysfunction of this system is linked to narcolepsy, as revealed in dogs with mutated HcrtR2 gene and mice lacking hypocretins or HcrtR2 (Lin et al., 1999; Willie et al., 2003). On the other hand, endocannabinoids are also involved in sleep regulation and have been shown to be strong sleep-inducers (Cravatt et al., 1995). Besides, systemic administration of rimonabant increases wakefulness and decreases REM sleep in rats (Santucci et al., 1996), whereas acute (Murillo-Rodríguez et al., 2001), subchronic (Herrera-Solís et al., 2010) and intrahippocampal (Rueda-Orozco et al., 2010) administration of anandamide has the opposite effect.

Few studies have investigated the possible mechanisms shared by the hypocretinergic and endocannabinoid systems in the regulation of sleep and wakefulness. The finding that cannabinoid signaling leads to hyperpolarization of hypocretin neurons and depolarization of MCH neurons in *in vitro* preparations (Huang et al., 2007) encouraged the idea that endocannabinoids could interact with these hypothalamic neurons to regulate sleep. Indeed, intra-hypothalamic administration of 2-AG increases REM sleep in rats through CB1 receptor and MCH signaling, since 2-AG slightly decreased c-fos expression in hypocretin neurons and activated MCH neurons (Pérez-Morales et al., 2013). Endocannabinoid and hypocretinergic systems could be involved in the modulation of sleep/wake cycle by acting in dorsal raphe nucleus (DRN) serotonergic neurons, closely linked to REM sleep and arousal. Thus, these neurons discharge at a high frequency during waking, at a lower rate during non-REM sleep and become silent during REM sleep (McGinty and Harper, 1976; Portas et al., 1996). Activation of HcrtRs increases activity of DRN serotonergic neurons (Brown et al., 2001; Liu et al., 2002). However, an electrophysiological study performed in rat DRN slices has reported that hypocretin-2 also inhibits glutamatergic transmission to serotonergic neurons of the DRN via retrograde endocannabinoid messengers (Haj-Dahmane and Shen, 2005). Although the functional implications of this retrograde synaptic modulation are not clear, the authors propose that it could prevent excessive excitation of DRN serotonergic neurons to provide a homeostatic control, contributing to the stable firing activity of these arousal-related neurons. Therefore, the loss of the hypocretin signal could lead to the disorganized activity and fragmented wakefulness observed in narcolepsy (Haj-Dahmane and Shen, 2005). However, further studies should be carried out to clarify the relevance of the possible interactions between cannabinoid and hypocretinergic systems in sleep modulation.

## Concluding remarks

The existence of a cross-talk between the hypocretinergic and endocannabinoid systems is strongly supported by their partially overlapping anatomical distribution and common role in several physiological and pathological processes. However, little is known about the mechanisms underlying this interaction. The formation of heteromers between HcrtR1 and CB1 receptors has been demonstrated *in vitro*, which alters the cellular localization and downstream signaling of both receptors. However, the biological significance of these heteromers remains unknown, and further studies are needed to verify whether the two receptors are expressed on the same target neurons and if they form heteromers *in vivo*. In this regard, better tools should be developed to determine the specific location of HcrtRs due to the cross-reactivity problems of the currently available antibodies (Kukkonen, 2012). On the other hand, hypocretin signaling has been reported to stimulate the synthesis of 2-AG leading to retrograde inhibition, which suggests that endocannabinoids might contribute to several hypocretin effects. Recent evidence denotes that this endocannabinoid-mediated retrograde inhibition is present in diverse brain regions *in vivo*, being of special relevance in the regulation of the analgesic effects induced by hypocretins. Interesting data also point to a collaboration of endocannabinoid and hypocretinergic systems in the central control of food intake and obesity. The hypocretinergic transmission is overstimulated in this pathological condition, and HcrtR antagonists might be useful in the control of appetite and other disorders associated with obesity, such as anxiety and sleep deregulations. However, the apparent antiobesity role of HcrtR2 in the peripheral control of energy balance should be taken into account and would possibly require the use of selective HcrtR1 antagonists for this specific purpose. Blockade of HcrtR1 signaling has demonstrated also its therapeutic potential against cannabinoid dependence by interfering with the rewarding effects of this drug. Nevertheless, the variety of hypocretin and endocannabinoid signaling implies that their manipulation to regulate a specific physiological process would probably produce several side effects. To avoid this problem, efforts should be focused on the development of selective agonists/antagonists for the different receptors and/or with site specific activity. Some authors defend that diverse GPCR heteromers are disease-specific and/or exhibit unique tissue specificity (Gomes et al., 2013). If this would be the case of CB1-HcrtR1 heteromers, they would serve as ideal drug targets with potentially lesser side effects that the single receptors. Although research about the cannabinoid-hypocretinergic interplay has only taken the first steps, future investigation in this field will lead to a better understanding of the therapeutic potential of this interesting interaction.

### Conflict of interest statement

The authors declare that the research was conducted in the absence of any commercial or financial relationships that could be construed as a potential conflict of interest.
